# Unconjugated secondary bile acids activate the unfolded protein response and induce golgi fragmentation via a src-kinase-dependant mechanism

**DOI:** 10.18632/oncotarget.13514

**Published:** 2016-11-23

**Authors:** Ruchika Sharma, Francis Quilty, John F. Gilmer, Aideen Long, Anne-Marie Byrne

**Affiliations:** ^1^ Department of Clinical Medicine, Institute of Molecular Medicine, Trinity Centre for Health Science, St James's Hospital, D08W9RT, Ireland; ^2^ School of Pharmacy and Pharmaceutical Sciences, Trinity College Dublin, Ireland

**Keywords:** bile acid, unfolded protein response, ER stress, golgi apparatus, oesophageal cancer

## Abstract

Bile acids are components of gastro-duodenal refluxate and regarded as causative agents in oesophageal disease but the precise mechanisms are unknown. Here we demonstrate that a specific subset of physiological bile acids affect the protein secretory pathway by inducing ER stress, activating the Unfolded Protein Response (UPR) and causing disassembly of the Golgi apparatus in oesophageal cells. Deoxycholic acid (DCA), Chemodeoxycholic acid (CDCA) and Lithocholic acid (LCA) activated the PERK arm of the UPR, via phosphorylation of eIF2α and up-regulation of ATF3, CHOP and BiP/GRP78. UPR activation by these bile acids is mechanistically linked with Golgi fragmentation, as modulating the UPR using a PERK inhibitor (GSK2606414) or salubrinal attenuated bile acid-induced effects on Golgi structure. Furthermore we demonstrate that DCA, CDCA and LA activate Src kinase and that inhibition of this kinase attenuated both bile acid-induced BiP/GRP78 expression and Golgi fragmentation. This study highlights a novel mechanism whereby environmental factors (bile acids) impact important cellular processes regulating cell homeostasis, including the UPR and Golgi structure, which may contribute to cancer progression in the oesophagus.

## INTRODUCTION

Oesophageal adenocarcinoma is a major source of cancer mortality and its incidence is increasing rapidly. Oesophageal adenocarcinoma is strongly associated with Barrett's Oesophagus, a pre-malignant metaplastic condition caused by persistent exposure of the oesophageal epithelium to gastro-duodenal refluxate [1]. These metaplastic cells undergo dysplasia and ultimately adenocarcinoma, a process known as the ‘Metaplsaia-dysplasia-adenocarcinoma sequence’ [2]. Bile acids present in gastro-duodenal refluxate are widely believed to contribute to the development of Oesophageal adenocarcinoma [3]. Evidence from *in vitro* and animal models implicate in particular, secondary bile acids, including deoxycholic acid (DCA) and lithocholic acid (LCA), their derivatives and chenodeoxycholic acid (CDCA) [3–5]. There are outstanding mechanistic questions regarding the influence of bile acids in this setting that are relevant to the development of chemoprevention strategies for patients with Barrett's Oesophagus.

The protein secretory pathway comprises protein biogenesis, processing, trafficking and secretion. Proteins are synthesised and processed through the Endoplasmic reticulum (ER) where they undergo folding, assembly and disulfide bond formation [6]. They then traffic to the Golgi apparatus for post-translational modification, glycosylation, and packaging for secretion. Extrinsic and intrinsic insults experienced by the cell, including nutrient deprivation and failure of post-translational modifications can lead to protein misfolding in the ER [7]. Accumulation of misfolded/unfolded proteins leads to ER stress and activation of the unfolded protein response (UPR). This involves dissociation of the chaperone protein BiP from PERK, ATF6 and IRE-1 allowing these proteins to initiate the UPR pathway in an effort to reduce ER burden as part of the ER stress recovery programme, to return the cell to normal protein homeostasis [6, 8]. The immediate response to ER stress is to decrease protein synthesis which is controlled by the PERK pathway. PERK monitors the balance between protein loading and protein folding capacity in the ER [7]. Under conditions of protein overloading, PERK attenuates protein translation by dimerising and activating eIF2α, the central regulator of protein synthesis. Increased expression of the chaperone proteins that aid protein folding (BiP, GRP94, Calreticulin and PDIs) facilitate recovery from the stress. When ER stress is alleviated, eIF2α is dephosphorylated and protein translation resumes. Whereas in normal cells unresolved UPR leads to pro-apoptotic signalling [7], tumour cells adapt to survive and circumvent apoptosis [9, 10]. Indeed tumour cells have increased trafficking and secretion of proteins by their very nature, to facilitate growth, angiogenesis and tumour-stroma interactions and they adapt the protein secretory pathway to meet these demands [11]. As part of the transformation process, cells undergo a “secretory switch” to provide the cell with increased secretory properties such as up-regulation of the chaperone protein BiP to allow for the increased demands of protein folding [12]. Increased BiP expression levels have been detected in various cancers including gastric cancers and plays a role in angiogenesis and tumour cell survival [10, 13]. Targeting the UPR has been suggested as a novel chemopreventative strategy for cancer [10].

Altered Golgi-associated processes such as protein glycosylation are also characteristics of cancer contributing to pro-survival and metastatic mechanisms [14]. Indeed morphological changes in the Golgi structure have been reported in multiple disease states, particularly neurodegenerative diseases [15]. We reported that fragmented Golgi structures are observed in biopsies of patients with colorectal and oesophageal cancer [16, 17]. Furthermore, the secondary bile acid DCA caused Golgi structure disassembly in colorectal and oesophageal cell lines (HCT116, HET1A, QH-tert, GO-tert, SKGT4) resulting in impaired post translational glycosylation, trafficking and secretion [16, 17]. Since aberrantly glycosylated/misfolded proteins would be trafficked back to the ER for re-processing, the aims of our study here were to investigate (i) the effect of a panel of bile acids present in the refluxate on Golgi structure, (ii) whether bile acids would cause ER stress and activate the UPR (iii) whether there was a mechanistic link between these two processes.

We report that a subset of bile acids perturb the protein secretory pathway causing Golgi fragmentation and activation of the PERK arm of the UPR. Furthermore, we identified a potential mechanistic link between these two processes that could be exploited as a novel chemopreventative/chemotherapeutic strategy for oesophageal cancer.

## RESULTS

### A select subset of bile acids activate the UPR in squamous oesophageal cells

**Figure 1:**
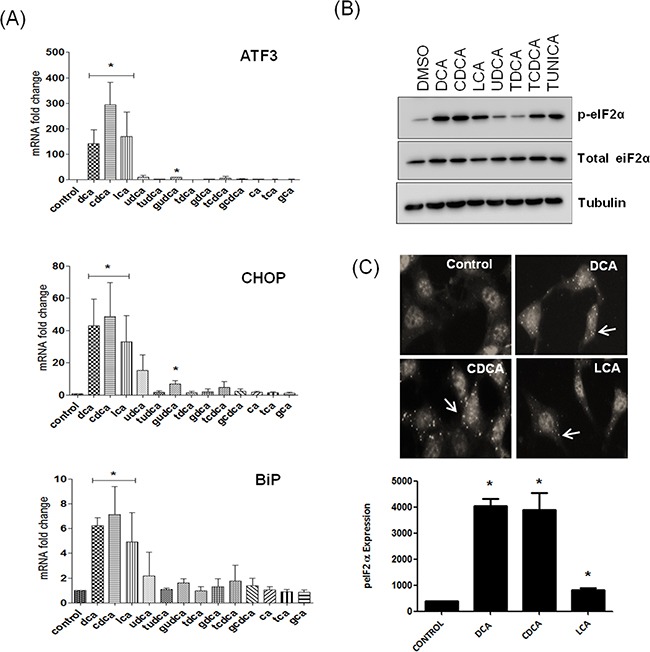
Bile acids activate the unfolded protein response in a selective manner **A.** HET-1A cells were treated with bile acids (300 μM) or LCA (25 μM) for 6 h and the effect of bile acids on AFT3, CHOP and BiP mRNA expression was quantified by Real-Time-PCR using GAPDH as denominator control gene. **B.** HET-1A cells were treated with bile acids (300 μM) or LCA (25 μM) for 6 h and the effect on eIF2α phosphorylation was measured by western blot. **C.** HET1A cells were treated with the unconjugated bile acids as indicated followed by immunofluorescence staining for phosphorylated eIF2α. Images were acquired using the InCell-1000 and quantified using the Investigator software package. Values represent the mean ± SEM for n=3 experiments, * p<0.05.

Exposure of the lower oesophagus to gastro-duodenal refluxate is considered to be the main contributory factor in promoting metaplasia, dysplasia and oesophageal adenocarcinoma. Gastric refluxate contains a mixture of acid, bile acids and pepsin. Acid was thought to be the main contributing factor to the development of Barrett's Oesophagus and adenocarcinoma but bile acids have since been proven to play a major role [3]. A panel of the bile acids typically found in gastro-duodenal refluxate were screened for their effects on the UPR in the non-neoplastic oesophageal cell line HET-1A. The panel consisted of DCA, CDCA, UDCA, CA, their glycine and taurine conjugates and LCA. Bile acids were screened at 300 μM (except LCA) as we previously demonstrated that this concentration does not lead to apoptosis in the 6 h time frame used during the experiment and LCA was tested at 25 μM, as we previously demonstrated that higher concentrations were cytotoxic [18]. These concentrations fall within the concentration range of bile acids found in the oesophagus in patients with the precancerous condition Barrett's metaplasia [19]. We initially screened the bile acid panel to investigate their effect on expression of UPR-associated genes ATF3, CHOP and BiP/GRP78. We demonstrate that DCA, CDCA and LCA increased expression of BiP (6 fold), CHOP (40-60 fold) and ATF3 (100-300 fold) (Figure 1A). The glycine or taurine-conjugated bile acids did not affect expression of these UPR genes with the exception of Glycoursodeoxycholic acid (GUDCA) which was the only conjugated bile acid to increase expression of ATF3 (9.7 fold) and CHOP (7 fold). UDCA was the only unconjugated bile acid that did not affect expression of BiP, CHOP or ATF3 (Figure 1A). None of the bile acids tested affected XBP1 expression (Supplementary Figure S2). These results demonstrate that a select subset of bile acids activate the UPR in oesophageal cells. As ATF3, CHOP and BiP mRNA expression are activated downstream of the PERK pathway we examined the effect of these bile acids on eIF2α activation. Phosphorylation of eIF2α is one of the first responses to ER stress as it switches off protein translation to relieve the burden on the ER. Treatment of HET-1A cells with DCA, CDCA and LCA increased levels of phosphorylated eIF-2α (Figure 1B, 1C). Neither UDCA nor tauroursodeoxycholic acid (TDCA) increased eIF2α phosphorylation (Figure 1B). Taurochenodeoxycholic acid (TCDCA) did increase phosphorylation but not to the same extent as the unconjugated form, CDCA (Figure 1B). These results indicate that a subset of the unconjugated bile acids (DCA, CDCA, LCA) activate eiF2α and that conjugated bile acids (with the exception of GUDCA) do not activate the eIF2α in oesophageal cells.

### Bile acid-activation of the UPR genes coincides with its effects on Golgi morphology

**Figure 2:**
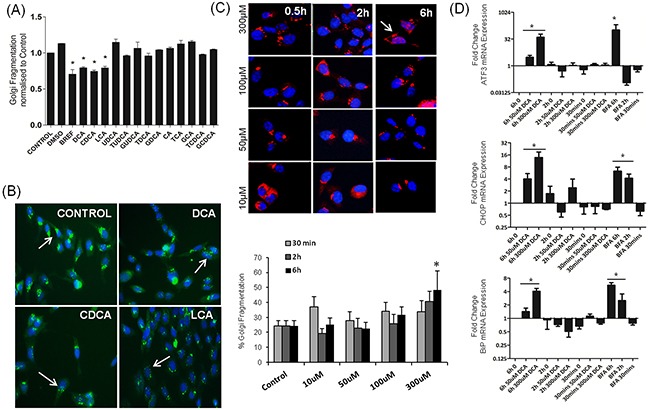
Bile acids alter Golgi morphology in a selective manner which correlates with UPR activation **A.** HET-1A cells were treated with bile acids (300 uM for all except LCA, 25 uM) for 6 h. Golgi morphology was identified using the GM130 antibody (green) and nuclei visualised using Hoechst (blue). Images were acquired using the InCell-1000 and quantified using the Investigator software package. **B.** Examples of images acquired using the InCell-1000 for bile acids indicated, original magnification 20×. **C.** HET1A cells were treated with DCA in a time and dose dependant manner as indicated. Golgi morphology was identified and quantified as above (GM130, red, Hoechst, blue). **D.** The time and dose dependence effect on mRNA expression levels of ATF3, CHOP and BiP was quantified by Real-Time PCR. Values represent the mean ± SEM for n=3 experiments, * p<0.05.

Previously we showed disassembly of the Golgi apparatus in response to DCA in a number of gastro-intestinal cell lines (HCT116, HET1A, QH-tert, GO-Tert, SKGT4) [16, 17]. Here we investigated the effects of a panel of bile acids on Golgi structure in HET-1A oesophageal cells and show that, of those tested, only DCA, CDCA and LCA induced Golgi fragmentation (Figure 2A, 2B). Treatment of HET-1A cells with UDCA, Cholic acid (CA) and their glycine and taurine conjugates did not induce Golgi fragmentation (Figure 2A). These results demonstrate that the bile acids that activate the UPR also cause Golgi fragmentation.

Having demonstrated that DCA, CDCA and LCA can induce Golgi Fragmentation and the UPR, we wanted to investigate if these two phenomena were connected. We next investigated the temporal relationship between UPR activation and Golgi fragmentation in response to DCA (50 uM, 300 uM) at 0.5 h, 2 h and 6 h. Golgi fragmentation was quantified as described in the materials and methods section and activation of the UPR was assessed by quantifying ATF3, CHOP and BiP mRNA expression. Both phenomena -Golgi fragmentation (Figure 2C) and UPR-associated gene activation (Figure 2D) were induced by 300 μM DCA at 6 h but not at earlier timepoints or at lower concentrations. Brefeldin-A (BFA), a known inducer of Golgi fragmentation, also increased mRNA expression of ATF3, CHOP and BiP (Figure 2D). These results suggest that UPR activation may be mechanistically linked to Golgi fragmentation.

### Blocking PERK pathway activation inhibits bile acid-induced Golgi fragmentation

**Figure 3:**
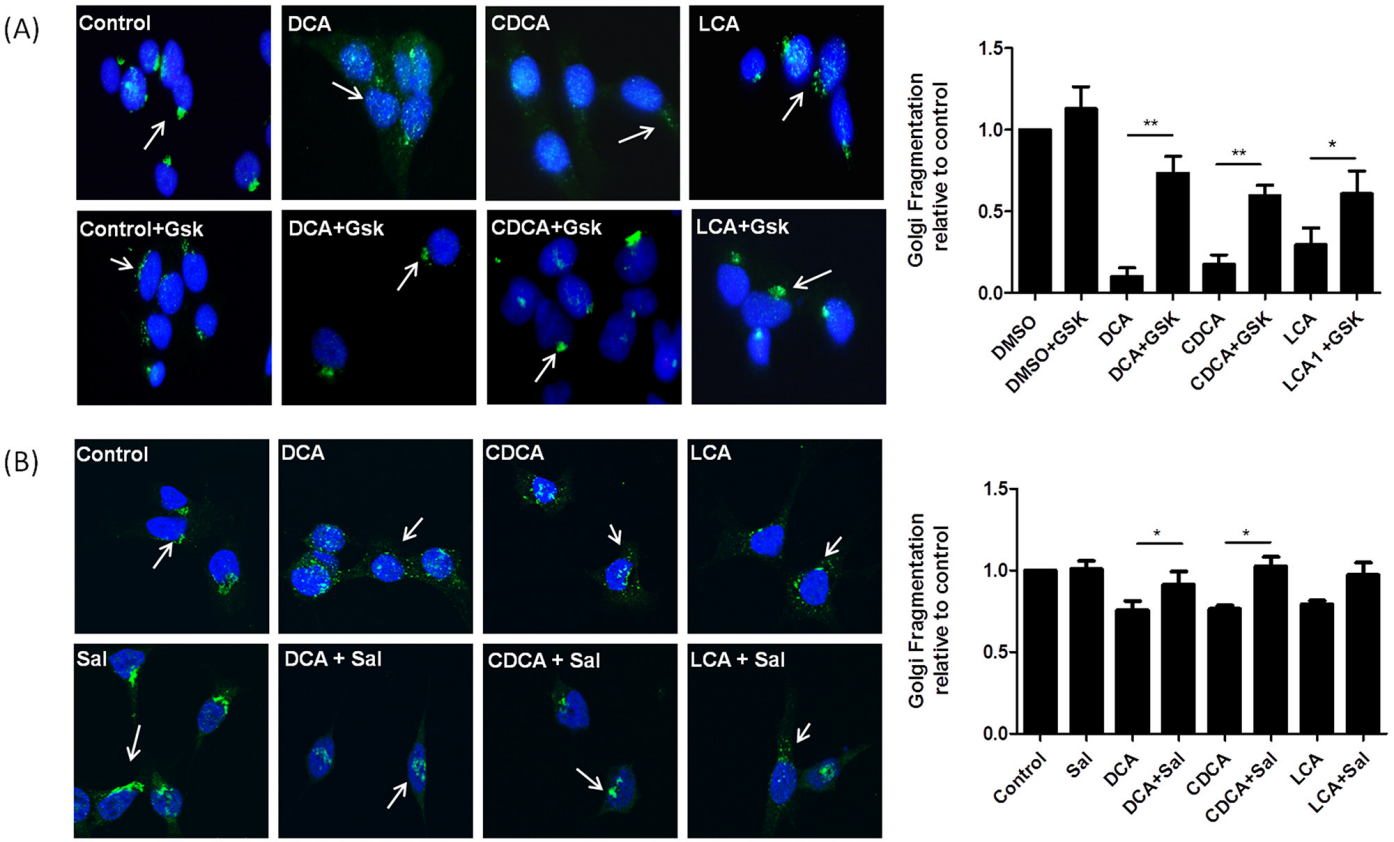
Blocking the PERK pathway attenuates Bile acid-induced Golgi Fragmentation **A.** HET-1A cells were pre-treated with GSK2606414 (Gsk, 1 μM) for 1 h then co-treated with GSK2606414 (1 μM) and DCA (300 μM), CDCA (300 μM) or LCA (25 μM) for a further 6 h. **B.** HET-1A cells were pre-treated with salubrinal (sal, 50 μM)for 18 h and then co-treated with salubrinal (50 μM) and DCA (300 μM), CDCA (300 μM) or LCA (25 μM) for a further 6 h. Cells were fixed, stained and images acquired using an LSM Zeiss confocal microscope and quantified as outlined in the materials and methods section. Data are represented as mean ± SEM, normalised to vehicle control for n=3 experiments, * p<0.05, ** p<0.005 versus bile acid without GSK or Salubrinal.

During ER stress BiP dissociates from PERK which autophosphorylates and activates eIF2α by phosphorylation. This causes a block in protein translation to decrease demand on the ER. GSK2606414 is an inhibitor of PERK phosphorylation, pre-treatment with this compound prevented bile acid-induced Golgi fragmentation (Figure 3A). Once ER stress is alleviated eIF2α is dephosphorylated and protein translation resumes. Salubrinal, an inhibitor of eIF-2α dephosphoylation, maintains this block in protein translation. When HET-1A cells were treated with salubrinal, no effect on the structure of the Golgi apparatus was observed (Figure 3B). However, pre-treatment of the cells with salubrinal decreased bile acid-induced Golgi fragmentation (Figure 3B). These results demonstrate that attenuating dephosphorylation of eIF-2α inhibits bile acid-mediated fragmentation of the Golgi apparatus providing further evidence of a relationship between ER stress/activation of the PERK pathway and Golgi morphology.

### Bile acids cause Golgi fragmentation and BiP activation via a Src-kinase dependant mechanism

c-Src is a proto-oncogene involved in cellular transformation. Src belongs to a large family of structurally-related protein tyrosine kinases with important functions in transducing signals from cell surface receptors influencing cell growth, survival, differentiation and migration [20]. Activation of Src is observed in several solid tumours and is also activated in high-grade-dysplasia and adenocarcinoma of the oesophagus [21, 22]. DCA treatment of colorectal cancer cells was previously shown to activate epithelial growth factor receptor (EGFR) and recruit Src to the cell membrane [23]. Activation of Src has been correlated with induction of Golgi fragmentation however the mechanism for this effect has yet to be elucidated [24]. Here we sought to determine if bile acids could activate Src in our model and if this was linked with bile-acid-induced Golgi fragmentation.

**Figure 4:**
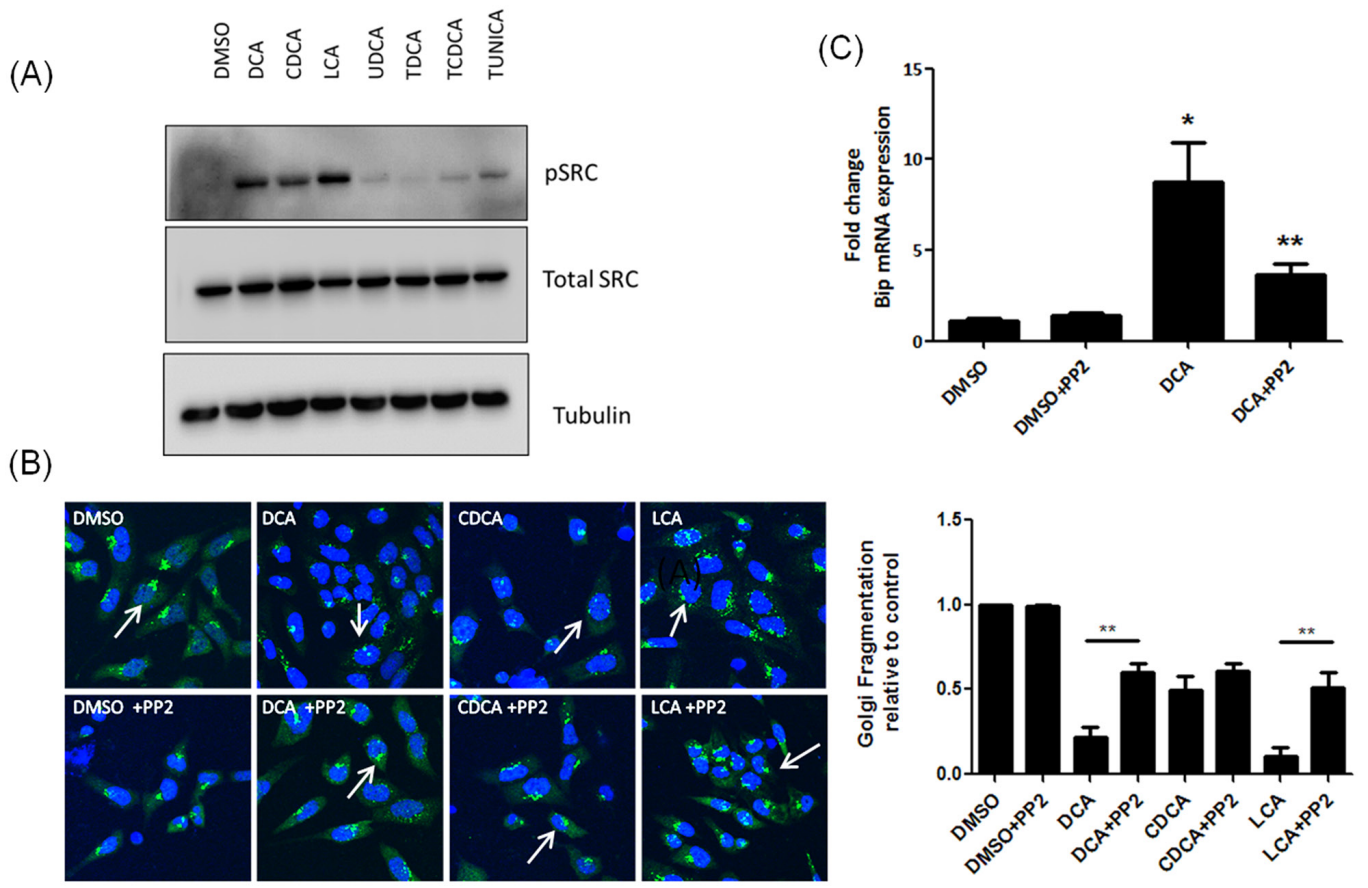
Unconjugated bile acids cause Golgi fragmentation and Bip activation via activation of Src **A.** HET-1A cells were treated with Bile acids and phosphorylation of Src was measured by western blot. **B.** Cells were pre-treated with PP2 (10 μM), prior to treatment with bile acids. Golgi structure was assessed using a GM130 antibody (green) and imaged using confocal microscopy as outlined in materials and methods. Nuclei were stained with Hoechst (blue). **C.** HET-1A cells were pre-treated with PP2 (10 μM) followed by treatment with DCA. Levels of Bip mRNA were quantified by RT-PCR. Data are represented as mean ± SEM, normalised to vehicle control for n=3 experiments, * p<0.05 versus Control **p<0.05 versus bile acid without PP2.

Src was activated only in response to those bile acids that induced Golgi fragmentation (DCA CDCA and LCA) (Figure 4A). Inhibition of Src activation by pre-treatment of cells with PP2 attenuated bile acid-induced Golgi fragmentation (Figure 4B). To investigate whether bile acid-activation of ER stress is also mediated via Src, we pre-treated the cells with PP2 prior to treating with DCA and quantified mRNA expression levels of BiP. Pre-treatment with PP2 attenuated DCA-induced mRNA expression of BiP. Increases in CHOP and ATF3 expression in response to DCA were not reduced by treatment with PP2 (Supplementary Figure S1). These results demonstrate that bile acid-mediated Golgi fragmentation and some elements of the bile acid-mediated UPR activation are regulated by Src kinase activity.

## DISCUSSION

There is mounting evidence that the protein secretory pathway is modulated in tumour cells during the process of tumourigenesis [11, 12]. Exposure to bile acids has been shown to cause ER stress and trigger the UPR in liver and intestinal cell lines and this has been linked to inflammation and cancer [25]. However the effects of bile acids in the oesophagus with respect to ER stress and other components of the protein secretory pathway, including the Golgi apparatus, has not been previously characterised. In this study, of the panel of bile acids tested, only DCA CDCA and LCA all activated eIF2α and caused Golgi fragmentation, whereas UDCA or taurine conjugated DCA or CDCA did not affect either process.

In normal cells, once ER homeostasis is restored the UPR is switched off, whereas under conditions of sustained ER stress, genes associated with apoptosis are induced [26, 27]. On the other hand, tumour cells with a sustained UPR in response to intrinsic or extrinsic stress, selectively modulate expression of genes that promote cell survival [11]. The PERK arm of the UPR involves phosphorylation of eIF2α which activates ATF4 and subsequently ATF3 to initiate transcription of genes involved in the UPR, including chaperones that aid protein folding (e.g BiP), amino acid metabolism and redox homeostasis. Exposure of oesophageal cells to DCA, CDCA or LCA resulted in eIF2α-phosphorylation and ATF3-CHOP-BiP expression. Examining the effect of bile acids on PERK and IRE-1 phosphorylation we found that all bile acids activated both PERK and IRE-1 (Supplementary Figure S1) suggesting that it is the activation of eIF2α that is crucial for the observed effects on Golgi fragmentation. Sustained activation of the PERK pathway is associated with tumourigenicity including the epithelial to mesenchymal transition (EMT). PERK−/− animals form smaller tumours and possess reduced angiogenesis capabilities [10]. BiP over-expression in tumours is linked with carcinogenesis and tumour cell survival; suppression of BiP in fibrosarcoma cells inhibited their ability to form tumours *in vivo* whereas increased BiP expression in glioma cells correlated with higher rates of proliferation [10].

Long-term exposure of a human colonic cell line (HCT116) to sublethal concentrations of DCA selected for cells resistant to DCA induced apoptosis [28]. These HCT116 colon cancer cells have increased expression of the BiP promoter, indicating that activation of the UPR provides a survival advantage for these cells [29]. Bip expression is also associated with Doxorubicin chemoresistance in human epidermoid carcinomas [30].

The impact of an altered Golgi morphology in the context of tumourigenesis is less well defined. Golgi fragmentation is observed as part of normal physiology, in cells undergoing mitosis and at end stage apoptosis [31]. However, we have shown inherent Golgi fragmentation in both colon and oesophageal cancer patient tissue [16, 17]. Moreover we demonstrated that the bile acid DCA causes dramatic disassembly of the Golgi [17]. This is not attributable to apoptosis, as DCA-induced Golgi fragmentation is a reversible process, upon removal of DCA (the stress), the Golgi structure reforms [17]. We previously published the consequences for bile acid-mediated Golgi fragmentation on the protein secretory pathway in terms of (i) secretion, which is blocked in response to DCA exposure (ii) N-linked protein glycosylation, which is impaired in response to DCA exposure and (iii) trafficking of E-cadherin to the cell membrane, which is reduced in response to DCA and Golgi fragmentation [16, 17]. The inherent fragmentation we observed in cancer tissue indicates the tumour cells can adapt to survive with a fragmented Golgi. The consequences for tumourigenesis include, glycosylation defects, which are considered a hallmark of cancer [16]. Indeed there are a number of colon cancer cell lines with constitutively fragmented Golgi including the SW480, Caco-2, HT-29 and T-84's which have abnormal glycosylation patterns [32]. A study investigating the impact of the microtubule network on protein trafficking show that disrupting the microtubule network leads to Golgi disassembly into mini-stacks, and after a period of adaptation these ministacks can glycosylate and transport cargo [33], although perhaps not as efficiently as cells with an intact Golgi structure. This adaptation process may be occurring in tumour cells. The question as to why the specific subset of bile acids (DCA, CDCA, LCA) perturbs the protein secretory pathway may relate to bile acid physiochemical properties and propensity to induce cell death in the HET-1A cell line. In a previous study we showed that DCA, CDCA and LCA significantly decreased HET-1A cell viability at 500 μM concentration [18]. In this study we show that, using sub-lethal concentrations of the panel of bile acids tested (300 μM), only DCA, CDCA and LCA activated eIF-2α in oesophageal cells and that only these bile acids caused Golgi fragmentation.

**Figure 5:**
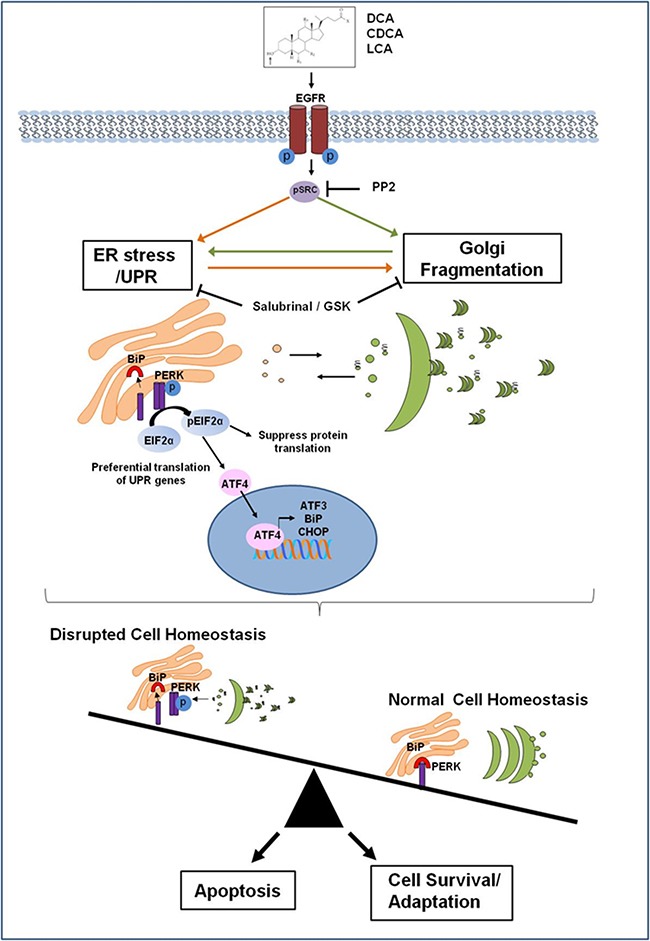
Proposed mechanism of how bile acids perturb the protein secretory pathway Bile acids activate the EGF receptor leading to activation of Src. This in turn activates the PERK pathway as part of the Unfolded Protein Response (UPR). BiP dissociates from PERK which dimerises and become phosphorylated which in turn phosphorylates eIF2α. This suppresses protein translation to reduce the burden on the ER, but also facilitates the translation of genes associated with ER stress recovery such as BiP, ATF3 and CHOP. DCA CDCA and LCA also induce Golgi fragmentation via activation of Src. Activation of the UPR may occur initially and subsequently cause Golgi fragmentation but once affected Golgi fragmentation would result in impaired protein glycosylation resulting in misfolded proteins that have to be sent back to the ER for re-processing. This could result in a sustained perturbance of the protein secretory pathway. Induction of ER stress could lead to Golgi fragmentation by either suppressing translation of a protein crucial for maintenance of Golgi structure via peIF2, or as a consequence of ER stress-induced apoptosis, if ER homeostasis is not restored. Bile acid disruption of normal cell homeostasis through their effects on the protein secretory pathway may lead to the selection of apoptotic resistant cell populations that have adapted to survive with a sustained UPR and fragmented Golgi. This suggests a novel mechanism for bile acid contribution to tumourigenesis, which could be exploited as novel therapeutic strategy.

The disruption of Golgi structure leads to impaired glycosylation processes, resulting in misfolded /misglycosylated proteins which would, in turn, be trafficked back to the ER for re-processing (Figure 5). The temporal relationship between Golgi fragmentation and UPR activation, together with the fact that the same bile acids at the same concentrations cause these effects, suggests that the effects of bile acids on these two components of the protein secretory pathway may be mechanistically linked. Indeed ATF3 and CHOP expression have previously been correlated with Golgi fragmentation in spinal motoneurons; neuronal cells expressing ATF3 or CHOP had fragmented Golgi whereas neurons not expressing ATF3 or CHOP had a normal Golgi structure [34]. To test this potential link in our model we targeted upstream regulators of Golgi morphology and the UPR.

Src kinase has been implicated in structure and function of the Golgi apparatus [24]. We first investigated if bile acid-induced Golgi fragmentation is mediated via a Src-kinase dependant mechanism. We show that of the panel of bile acids studied, only the bile acids that caused Golgi Fragmentation (DCA, CDCA and LCA) activate Src (Figure 4A). The Src inhibitor PP2 decreased DCA- induced Golgi fragmentation (Figure 4B). Importantly, PP2 also decreased DCA-induced BiP expression demonstrating a link between Golgi fragmentation and the UPR. PP2 did not attenuate bile acid-induced ATF3 or ATF4 expression suggesting other mechanisms are involved (Supplementary Figure S3). BiP mRNA expression can be up regulated independently of PERK [27], therefore Src may activate transcriptional regulation of BiP independent of ATF3 and ATF4. Src is a proto-oncogene that interacts with many transmembrane receptors regulating downstream cell signalling cascades that promote cell survival and tumourigenesis [35]. Src is crucial for tumour cell metastasis by regulating the cell cytoskeleton, facilitating breakdown of tight junctions through activation of p120-catenin and decreasing adhesion to the extracellular matrix through regulating focal adhesion complexes providing a further pro-tumourigenic role for these bile acids in the oesophagus. Tyrosine phosphorylation events are key in the regulation of ER stress. PERK, a dual specificity kinase, is itself phosphorylated on multiple tyrosine residues with both positive and negative effects [36, 37]. The protein tyrosine phosphatase, PTP-1B, potentiates IRE1 signalling during ER stress [38]. While these proteins have not been shown to be direct substrates of Src, this enzyme is central to the regulation of ER stress in many systems. For example, Fan et al [39] demonstrate that Src acts as an adaptor protein for the Estrogen Receptor in its activation of stress responses induced by oestrogen in MCF-7-5C cells. They demonstrated that oestrogen activated Src through the Oestrogen Receptor and inhibition of Src abolished the phosphorylation of eIF2α in these cells. We demonstrate that inhibition of Src activity attenuates bile acid-induced Golgi fragmentation and ER stress (BiP activation). DCA was previously demonstrated to activate EGFR and recruit Src to the cell membrane indicating Src is activated upstream of these processes [23] (Figure 5).

Salubrinal is a compound that has been shown to protect cells from ER stress-induced apoptosis by inhibiting eIF2α dephosphorylation [40, 41]. Further evidence of a mechanistic link between Golgi fragmentation and the UPR in response to bile acids is provided by the fact that pre-treatment of cells with salubrinal attenuates bile acid-induced Golgi fragmentation (Figure 3). There are at least two possible explanations why salubrinal attenuates bile acid-induced Golgi fragmentation. Firstly, in the case for apoptosis, CHOP activated by DCA CDCA and LCA would allow dephosphorylation of eIF2α and translation of pro-apoptotic proteins and thus initiation of Golgi fragmentation. [40]. The second possible explanation is that, by maintaining the translational block, salubrinal could prevent the translation of a protein/signal that causes Golgi disassembly. Hence activation of the UPR leads to Golgi fragmentation, abnormal glycosylation and altered protein secretion. This in turn could further amplify the UPR, thus establishing cycles of aberrant signalling in apoptosis resistant populations. As we demonstrated bile-acid activation of PERK at an earlier timepoint (1 h, Supplementary Figure S1) compared to the timepoint we observe fragmentation of the Golgi (6 h), could indicate that ER stress occurs first. However, we must also consider that Golgi fragmentation could occur first, as the Golgi has previously been identified as a sensor of stress [42]. Hicks and Machamer suggest that morphological changes in Golgi structure could transduce a stress signal and activate the UPR leading to apoptosis or cell survival [42]. In either circumstance it is evident that bile acids perturb the protein secretory pathway affecting both Golgi structure and activation of the UPR. It is not yet clear if mutageneis or altered Golgi-associated gene expression contributes to tumourigenesis, however we demonstrated that a Golgi-associated protein GOLPH2 is cleaved and secreted in response to DCA [43]. This cleaved GOLPH2 facilitates tumour cell invasion implicating a potential link between altered Golgi-associated protein expression and invasion/metastasis.

The balance between survival and apoptosis relies on the signalling pathways activated downstream of these processes (Figure 5). Apoptotic resistant cells with persistent UPR activity and altered Golgi morphology likely promote tumourigenesis in a multi-factorial fashion.

In conclusion, this study established a novel mechanism of bile acid-mediated effects on the ER and Golgi components of the protein secretory pathway, which may contribute to the carcinogenic cascades driving malignant transformation in oesophageal carcinogenesis. Therapeutic targeting of the UPR has been suggested, by either inhibiting the pro-survival signals of the UPR so that tumour cells cannot adapt to the stressful conditions or else to manipulate/target the UPR to promote apoptosis. In the context of oesophageal cancer this approach could be employed as a therapeutic for established tumours with an elevated UPR. To prevent disruption of the protein secretory pathway, we provide a rationale for using an anti-bile acid therapeutic as a chemopreventative strategy for patients with pre-malignant oesophageal disease.

## MATERIALS AND METHODS

### Chemicals

Bile acids were obtained from Sigma-Aldrich Chemical Co. (St. Louis, MO, USA), maintained as 200 mM stock solutions in DMSO and diluted as required with medium. Salubrinal was purchased from Calbiochem, (Nottingham, UK). PP2 was obtained from Sigma-Aldrich Chemical Co. (St. Louis, MO, USA), and GSK2606414 was obtained from Merck Millipore (Darmstadt, Germany).

### Cell culture

The human oesophageal squamous epithelial cell line HET-1A, was obtained from American Type Culture Collection (ATCC, Rockville, MD). HET-1A cells were cultured in bronchial epithelial basal medium (Lonza Group Ltd, Switzerland) supplemented with triiodothyronine, insulin, transferrin, retinoic acid, hydrocortisone, human recombinant epidermal growth factor, epinephrine and bovine pituitary extract according to manufacturers instructions. Cultures were maintained at 37°C in a humidified atmosphere containing 5% CO_2_.

### RNA extraction and real time reverse transcription-polymerase chain reaction (RTPCR)

HET-1A cells were seeded at 2 × 10^5^ cells/T-25 cm^2^ flask and treated with bile acids as indicated. Cellular RNA was extracted using spin column purification (Machery-Nagel, Düren, Germany). RNA was transcribed to cDNA using the Verso^TM^ SYBR^®^ Green 2-step QRT-PCR Rox kit (Thermofisher Scientific, Waltham, USA). PCR reactions were performed in the ABI Prism 7900HT thermocycler (Applied Biosystems, Foster City, CA) using primers listed in supplementary Table S1. Fold inductions were calculated using the comparative C_T_ method as outlined in ABI prism manual with GAPDH as denominator control gene. Values represent the mean ± SEM for n=3 experiments.

### Western blot for ER stress markers

HET1A cells were treated with bile acids for 6 h. For PP2 pre-treatment studies PP2 (10 uM) was added 24 h prior to co-treatment with bile acids for 6 h. For salubrinal and GSK2606414 pre-treatment studies, these compounds were added 1 h prior to co-treatment with bile acids. Cells were lysed with NP40 buffer and protein quantified using a Pierce^TM^ BCA kit (Thermo Fisher Scientific Waltham, MA USA). Equal concentrations of protein were separated by SDS-PAGE and transferred to PVDF membranes. After blocking with 5% Marvel, primary antibodies; pPERK/Total PERK/total IRE-1/pSrc/total src/pEFI2a/total EIF2α/tubulin/actin (Cell signalling Technologies Inc. Danvers, MA, USA) or pIRE-1 (Abcam, Cambridge, United Kingdom) were incubated overnight in 5% BSA/TBST. HRP-conjugated secondary antibodies (Santa Cruz Biotechnology, Inc. Dallas, Texas) were incubated for 1 h and detected using Pierce^TM^ ECL substrate (Thermo Fisher Scientific, Waltham, MA USA).

### Immunofluorescence

Cells were plated into 96-well plates (10 × 10^3^ cells/ml, 100μL/well). After 48 h cells were treated with bile acids for 6 h in complete medium. Control wells were treated with complete medium or 0.15 % DMSO as a vehicle control. Cells were fixed, permeabilised, blocked using 5 % Bovine Serum Albumin (Sigma-aldrich Chemical Co. St. Louis, MO, USA), and incubated with anti-peIF2α antibody (Cell signalling Technologies Inc. Danvers, MA, USA) for 1 h followed by AlexaFluor-488-conjugated secondary antibody for 30 min (Invitrogen, Carlsbad, CA, USA). Images were acquired by High Content Analysis using the GE Incell-1000 (GE Healthcare, Little Chalfont, UK). Six fields of view per well were acquired in duplicate wells for n=3 experiments, original magnification 20×. The intensity of phosphorylated eIF-2α was quantified using the Investigator software package (GE Healthcare, Little Chalfont, UK). Punctate cytoplasmic structures of phospho-eIF-2α were quantified by quantifying the fluorescence intensity and total area of the punctuate structures. High Content Analysis using the GE Incell-1000 (GE Healthcare, Little Chalfont, UK). Reported values are expressed based on Intensity x Total area.

### Assessment of golgi morphology

Cells were plated into 96-well plates (8 × 10^4^ cells/ml, 100 μL/well). After 24 h cells were pre-treated with salubrinal 50 μM in. After a further 18 h cells were co-treated with bile acids and salubrinal or bile acids alone for another 6 h. Control wells were treated with supplement free medium, 0.5% DMSO or Brefeldin-A (1 μg/ml) as positive control for Golgi fragmentation. Cells were fixed, permeabilised with 0.3% Triton-X, blocked with 5% BSA and incubated with anti-GM130 antibody (all from Sigma-Aldrich Chemical Co., St Louis, MO) for 1h followed by AlexaFluor-488-conjugated secondary antibody for 30 min (Invitrogen, Carlsbad, CA, USA). Images were acquired using the GE Incell-1000 (GE Healthcare, Little Chalfont, UK). Six fields of view per well were acquired in duplicate wells for n=3 experiments, original magnification 20×. Golgi fragmentation was quantified using the Investigator software package (GE Healthcare, Little Chalfont, UK). The multi-target analysis algorithm was optimised to detect Golgi fragments within cells. The lower the mean area was used to depict smaller Golgi fragments within the cell. A cut-off for mean Golgi area in control wells was incorporated in to the algorithm and classified as intact Golgi. Cells containing Golgi with a mean area lower than this cut off were classified as fragmented. The % number of cells with fragmented Golgi was reported relative to control. This methodology was used for experiments in Figure 2 and Figure 3B. Due to unavailability of the Incell-1000 performing other experiments, Golgi fragmentation was assessed using images acquired by confocal microscopy. At least 5 fields of view were obtained per treatment, in triplicate, to obtain 200 cells per treatment and then classified as either intact or fragmented. The % Golgi fragmentation was then normalised to vehicle control. This methodology was used for Figure 3A and Figure 4B.

### Statistics

Statistical analysis was assessed using paired Students T-tests or Anova with Dunnet's post-hoc correction analysis as appropriate with Graphpad Prism 5. Data are represented as means ± standard error of the mean (SEM) for n=3 experiments.

## SUPPLEMENTARY FIGURES AND TABLE



## Abbreviations

UPR: Unfolded Protein Response
DCA: Deoxycholic acid
CDCA: Chenodeoxycholic acid
LCA: Lithocholic acid
UDCA: Ursodeoxycholic acid
TUDCA: Taurodeoxycholic acid
GUDCA: Glycoursodeoxycholic acid
TDCA: tauroursodeoxycholic acid
GDCA: Glycodeoxycholic acid
TCDCA: Taurochenodeoxycholic acid
GCDCA: Glycochenodeoxycholic acid
CA: Cholic acid
TCA: Taurocholic acid
GCA: Glycocholic acid
ER: Endoplasmic reticulum
BFA: Brefeldin-A
EGFR: Epithelial growth factor receptor
EMT: Epithelial to mesenchymal transition
SEM: Standard error of the mean
