# Supplementary material for: Unconjugated secondary bile acids activate the unfolded protein response and induce golgi fragmentation via a src-kinase-dependant mechanism
Source: Oncotarget. 2016 Nov 23;8(1):967–78. doi: 10.18632/oncotarget.13514 (PMC5352210; doi:10.18632/oncotarget.13514)
Supplement: Supplementary file 1 [file oncotarget-08-967-s001.pdf]

Unconjugated secondary bile acids activate the unfolded protein response and induce Golgi fragmentation via a src-kinase-dependant mechanism

SUPPLEMENTARY FIGURES AND TABLE

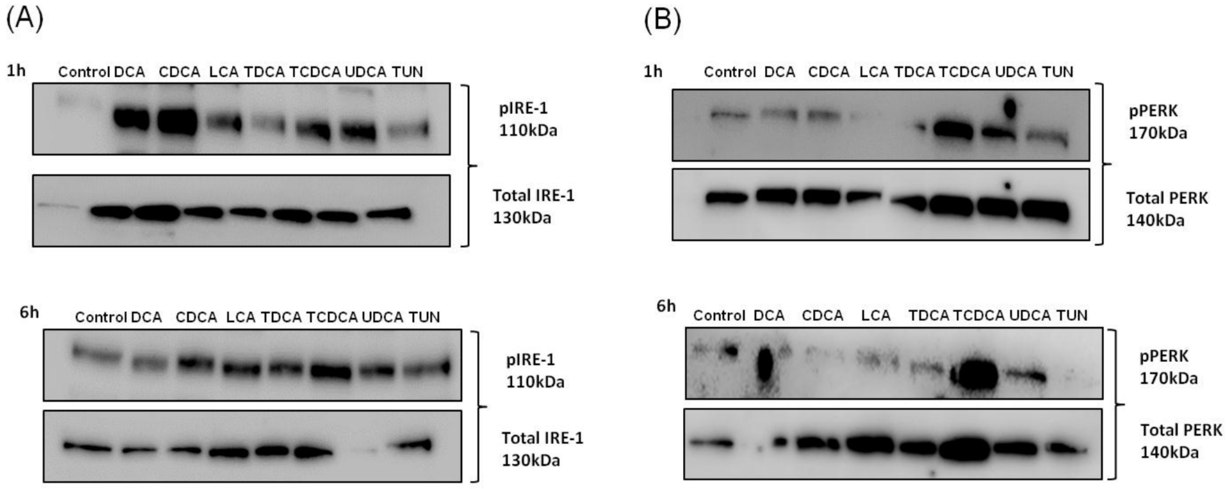

Supplementary Figure S1: Bile acid activation of IRE-1 and PERK. HET-1A cells were treated with bile acids (300 µM) or LCA (25 µM) or 1 µg/ml Tunicamycin (TUN) for 1 h or 6 h and the effect on IRE-1 and PERK phosphorylation was measured by western blot. All bile acids activated IRE-1 at both 1 h and 6 h timepoints. All bile acids activated PERK at 1 h but basal levels were also observed in control cells. Activation of PERK was barely detected at 6 h. Images are representative of n=3 experiments.

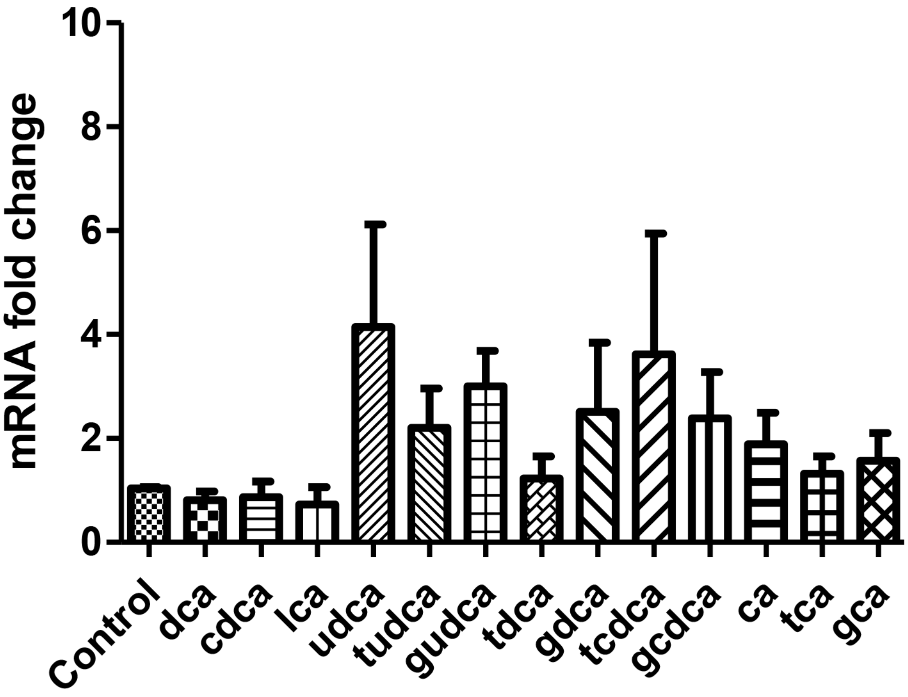

Supplementary Figure S2: Bile acids do not significantly alter XBP1 mRNA expression. HET-1A cells were treated with bile acids (300 µM) or LCA (25 µM) for 6 h and the effect of bile acids on XBP1 mRNA expression was quantified by Real-Time-PCR using GAPDH as denominator control gene. Values represent the mean ± SEM for n=3 experiments.

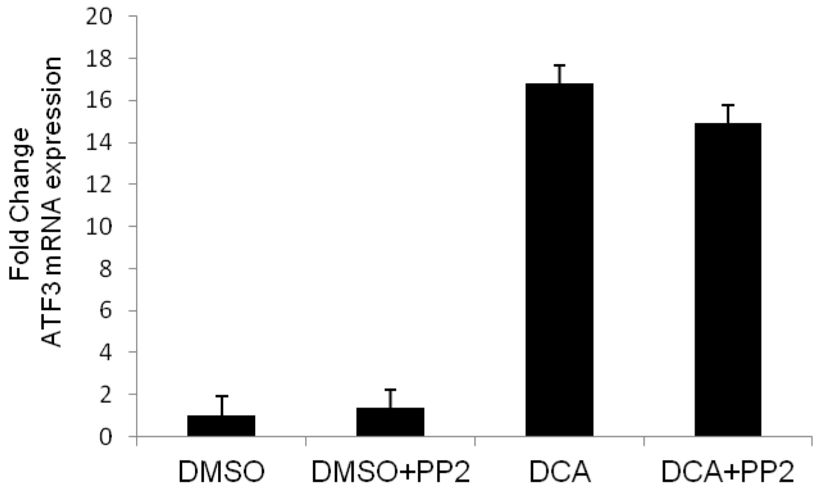

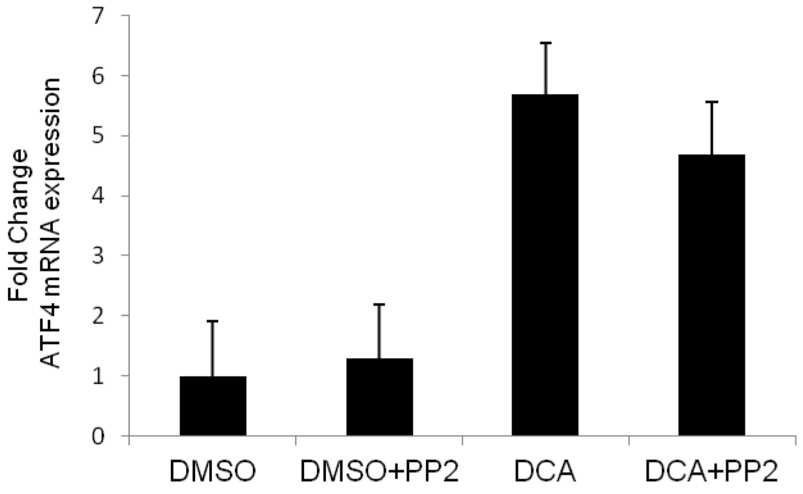

Supplementary Figure S3: PP2, does not attenuate DCA-induced ATF3 or ATF4. HET-1A cells were pre-treated with PP2 (10 µM) followed by treatment with DCA (300 µM) for 6 h. Levels of ATF3 or ATF4 mRNA were quantified by RT-PCR. Data are represented as mean ± SEM, normalised to vehicle control for n=3 experiments, * $p<0.05$ versus Control ** $p<0.05$ versus bile acid without PP2.

Supplementary Table S1: Taqman® gene expression assay primers used for real-time pcr in this study. All primers were obtained from applied biosystems. Gene names highlighted in bold are those referred to in the manuscript.

Applied biosystems code	Gene name	Alias
Hs00358796_ml	DDIT3	CEBPZ, **CHOP**, CHOP-10, CHOP10, GADD153
Hs02856596_ml	**XBP-1**	TREB5, XBP-1, XBP2
Hs99999174_ml	HSPA5	**Bip**/Grp78/MIF2
Hs00231069_ml	**ATF3**	RP11-338C15.1
Hs00909569_gl	**ATF4**	CREB-2, CREB2, TAXREB67, TXREB
Hs99999905_ml	**GAPDH**	CDABP0047, G3PD, GAPD
